# Avulsion Fracture of the Coracoid Process at the Coracoclavicular Ligament Insertion: A Report of Three Cases

**DOI:** 10.1155/2016/1836070

**Published:** 2016-11-22

**Authors:** Takeshi Morioka, Kiyohisa Ogawa, Masaaki Takahashi

**Affiliations:** ^1^Department of Orthopedic Surgery, Eiju General Hospital, 2-23-16 Higashi-Ueno, Taito-ku, Tokyo 110-8645, Japan; ^2^Department of Orthopedic Surgery, National Hospital Organization Tokyo Medical Center, 2-5-1 Higashigaoka, Meguro-ku, Tokyo 152-8902, Japan

## Abstract

Avulsion fracture at the site of attachment of the coracoid process of the coracoclavicular ligament (CCL) is extremely rare. We presented three adult cases of this unusual avulsion fracture associated with other injuries. Case  1 was a 25-year-old right-handed male with a left distal clavicular fracture with an avulsion fracture of the coracoid attachment of the CCL; this case was treated surgically and achieved an excellent outcome. Case  2 was a 39-year-old right-handed male with dislocation of the left acromioclavicular joint with two avulsion fractures: one at the posteromedial surface of the coracoid process at the attachment of the conoid ligament and one at the inferior surface of the clavicle at the attachment site of the trapezoid ligament; this case was treated conservatively, and unfavorable symptoms such as dull pain at rest and sharp pain during some daily activities remained. Case  3 was a 41-year-old right-handed female with a right distal clavicular fracture with an avulsion fracture of the coracoid attachment of the conoid ligament; this case was treated conservatively, and the distal clavicular fracture became typical nonunion. These three cases corresponded to type I fractures according to Ogawa's classification as the firm scapuloclavicular connection was destroyed and also to double disruption of the superior shoulder suspensory complex. We recommend surgical intervention when treating patients with this type of acute or subacute injury, especially in those engaging in heavy lifting or overhead work.

## 1. Introduction

The coracoid process can fracture at various sites [1, 2]. However, avulsion fracture at the site where the coracoclavicular ligament (CCL) attaches to the coracoid process is very rare; an extensive literature search found only six such cases [3–8]. Four of these cases were adolescents in whom avulsion fracture of the coracoid apophysis attached by the CCL occurred [5–7]. Fracture of three adolescent cases was associated with complete acromioclavicular separation [5, 6]. Presented here are three cases of adults with similar unusual avulsion fractures associated with other injuries including acromioclavicular joint (ACJ) dislocation and distal clavicular fracture. These three patients were provided with a written explanation of the importance of the publication of their cases and gave their consent.

## 2. Case Presentation

### 2.1. Case  1

A 25-year-old right-handed male office worker became inebriated with a friend at a social function and they fell together, with his friend landing on top of his left shoulder. The patient experienced severe left shoulder pain soon after the fall, visited a local orthopedic clinic on the day of the injury, and was referred to our hospital the next day.

**Figure 1:**
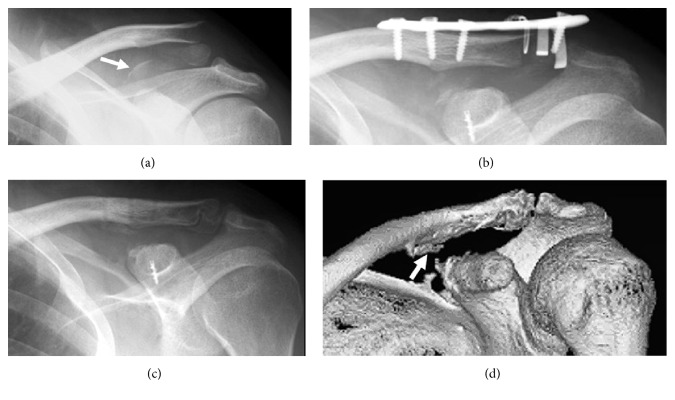
Imaging of Case  1. (a) Radiogram taken at the first visit showing distal clavicular fracture and avulsion fracture of the upper side of the coracoid process (arrow). (b) Postoperative radiogram revealing the well-reduced clavicular fracture and the fractured coracoid fragment. (c) Roentgenogram taken 1 year postoperatively indicating firm bony union of the distal clavicular and coracoid process fractures. (d) Three-dimensional computed tomography performed 1 year postoperatively demonstrating thin new bone formation at the conoid tubercle (arrow).

Examination showed swelling around the left distal clavicle and shoulder pain that increased with elevation of the shoulder. Past medical and family histories were noncontributory. Radiography demonstrated a left distal clavicular fracture (type 2b according to Craig's classification and type 3B1 according to Robinson's classification) with an avulsion fracture of the coracoid attachment of the CCL [9] (Figure 1(a)).

Surgery was performed on the third day after the injury via a horizontal incision just above the distal clavicle. The conoid ligament had been avulsed from the coracoid with a bone fragment of approximately 15 mm × 10 mm. A small part of the trapezoid ligament had also been avulsed with another small bone fragment; however, most of the trapezoid ligament was anatomically intact. A Scorpion® plate (Aimedic MMT, Tokyo, Japan) was used to secure the fractured distal clavicle. The conoid ligament with avulsed bone fragment was fixed to the fracture bed of the coracoid with a Ti Screw Suture Anchor with EasySlide™ Surface Treatment (Biomet, Warsaw, Indiana, USA) (Figure 1(b)).

After 3 days of immobilization in a sling, passive range of motion exercise of the shoulder was started. Active range of motion exercise was initiated at postoperative week 6. The Scorpion plate was removed at 8 months postoperatively. The fractured bones had achieved a firm union 1 year postoperatively (Figures 1(c) and 1(d)). The Constant score ratio to the normal side was 100% at 2 years postoperatively [10].

### 2.2. Case  2

A 39-year-old right-handed male journal editor was hit by a car while walking. He was taken by ambulance to the emergency room of our hospital, where a physician diagnosed traumatic subarachnoid hemorrhage and anterior cruciate ligament injury of the right knee. The patient required hospitalization, during which he complained of persistent left shoulder pain. He was referred to us 3 weeks after the initial injury.

**Figure 2:**
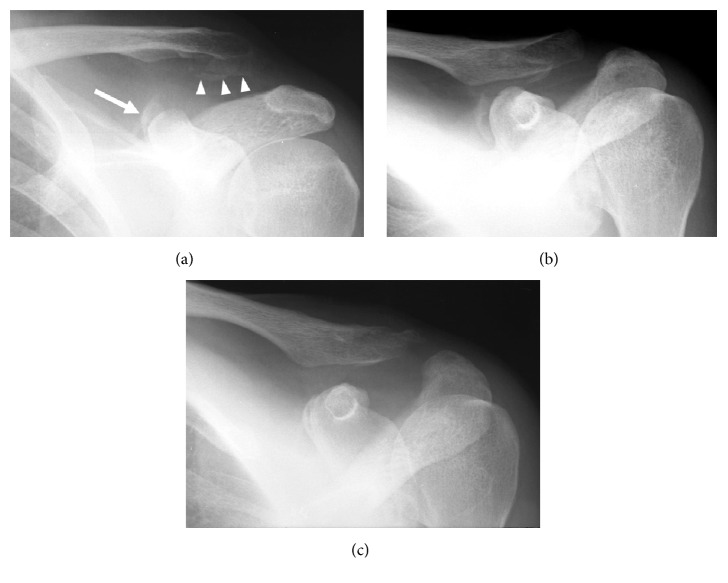
Imaging of Case  2. (a) Radiogram taken at the first visit showing the acromioclavicular dislocation and avulsion fracture of the superomedial side of the coracoid process (arrow) and the inferior side of the clavicle (arrow heads). (b) Angle up view taken at the first visit showing avulsion fracture of the coracoid located on the posteromedial side of the coracoid angle (compatible with the attaching site of the conoid ligament). (c) Radiogram taken 1 year and 6 months after the injury demonstrating firm union of the avulsed coracoid fragment.

Examination revealed tenderness and swelling surrounding the ACJ. The left shoulder was painful on active movement, although its active and passive ranges of motion were not limited. Past medical and family histories were noncontributory. Radiographs showed dislocation of the left ACJ (type III according to Rockwood's classification) with fractures of the posteromedial surface of the coracoid process and of the inferior surface of the clavicle [11] (Figures 2(a) and 2(b)). The attachment site of the trapezoid ligament to the clavicle and that of the conoid ligament to the coracoid (compatible with the posteromedial side of the coracoid angle) appeared to be avulsed; hence, we assumed that the CCL function was lost. As 3 weeks had already elapsed since the injury, we initiated conservative therapy with a sling for 3 weeks.

Although bony union of the avulsion fractures was confirmed on the radiograms taken 1 year and 6 months after the injury, the coracoclavicular interval and ACJ space remained wide (Figure 2(c)). At 3 years and 6 months after the injury, physical examination showed mild posterior instability and superior protrusion of the distal end of the clavicle. The stress test for the ACJ was negative. The patient complained of dull pain at rest and sharp pain when downward traction was placed upon the arm or when he engaged in overhead work. The Constant score ratio was 88% [10].

### 2.3. Case  3

A 41-year-old right-handed woman without occupation who suffered from osteoporosis due to anorexia nervosa fell on her right shoulder. She experienced severe right shoulder pain soon after the fall and presented at our orthopedic clinic on the day of the injury. She had undergone left femoral prosthetic replacement due to traumatic femoral neck fracture 3.5 months previously.

**Figure 3:**
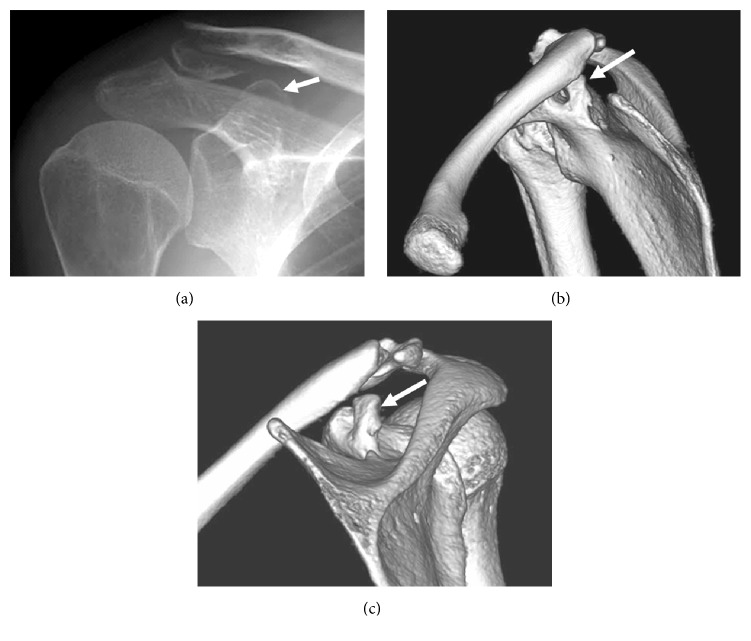
Imaging of Case  3. (a) Radiogram taken at the first visit revealing distal clavicular fracture (type 2b according to Craig's classification) and avulsion fracture of the posterior side of the coracoid process (arrow). (b and c) Three-dimensional computed tomography taken 1 year after the injury demonstrating nonunion of the lateral clavicular fracture and the malunited coracoid fragment migrating posterosuperiorly (arrow).

Examination revealed swelling around the left distal clavicle and shoulder pain that increased with shoulder movement. Radiography showed a right distal clavicular fracture (type 2b according to Craig's classification [9]) with an avulsion fracture of the coracoid attachment of the conoid ligament (Figure 3(a)). We recommended surgical treatment because of the probability that the CCL function was lost; however, the patient refused surgery.

After 3-month immobilization using a figure-of-eight splint and passive range of motion exercise of the shoulder was started. One month later, active shoulder ROM was begun, with some dull pain during movement. Five months after the initial shoulder injury, the patient was in a traffic accident that caused subdural hematoma with resultant right hemiplegia and aphasia. Hence, functional evaluation was impossible 1 year after the shoulder injury. Although bony union of the avulsion fracture of the coracoid was confirmed by computed tomography (CT), the distal clavicular fracture showed typical nonunion (Figures 3(b) and 3(c)).

## 3. Discussion

Fracture of the coracoid process occurs more frequently than previously thought and is well known to occur in various anatomical sites [1, 2]. Although coracoid fractures in adults have been classified into four or five types based on their anatomical position, Ogawa et al. [12] classified coracoid fractures into two types from a functional point of view according to the relationship between the fracture site and the CCL; type I fractures occurring proximal to this ligament attachment destroy the scapuloclavicular connection, whereas type II fractures distal to this attachment basically preserve this connection. The cases presented above corresponded to type I fractures, as the scapuloclavicular connection had been destroyed. According to the characteristic fracture site, they should be considered as a subtype of type I fractures.

Nearly all type I fractures are complex injuries and are consistent with the double disruption of the superior shoulder suspensory complex (SSSC) proposed by Goss [13, 14]. There is no consensus regarding the typical mechanism of injury that produces type I fracture [14]. However, a number of reported cases had a common mechanism of injury that was clearly caused by force exerted on the lateral aspect of the shoulder [1, 2, 12–17]. This mechanism is consistent with the concept of lateral impaction injuries, in which a powerful impaction force exerted on the lateral aspect of the shoulder is transmitted via the humeral head to the scapula and clavicle to cause a range of injuries [18]. The mechanism of injury in our cases seems to match this concept. The immediate cause of type I fractures in the majority of cases, however, is unquestionably traction caused by the CCL associated with concurrent injuries, particularly ACJ dislocation, and distal clavicular or acromial fractures [14]. When the traction force is applied to the CCL, breakdown may occur in four different locations and forms: avulsion fracture of the CCL attachment to the clavicle, tear of the CCL itself, avulsion fracture of the CCL attachment to the coracoid process, and/or type I coracoid fracture. The site of the damage is determined by the relative mechanical strength of the bone and ligament, as well as the amount and direction of the applied force. The exact mechanism of injury producing avulsion fracture of the CCL attachment to the coracoid process remains unknown.

Our three cases correspond to double disruption of the SSSC, in which surgical treatment is indicated in acute cases [13]. The first case was treated surgically and had an excellent outcome. The second and third cases were treated conservatively, and the outcomes were not favorable. The second case still had unfavorable symptoms such as dull pain at rest and sharp pain while applying downward traction or engaging in overhead work 3 years and 6 months after the injury. The third case could not be functionally evaluated 1 year postoperatively, but CT showed nonunion of the distal clavicular fracture. A previously reported case of an adolescent treated conservatively also resulted in some aching during activity [5]. We therefore believe that surgical intervention, including precise repositioning of the fractured fragment and appropriate stabilization of associated injuries, should be considered when treating patients with this type of acute injury, especially for those engaging in heavy lifting or overhead work.
